# Intramolecular Cohesion of Coils Mediated by Phenylalanine–Glycine Motifs in the Natively Unfolded Domain of a Nucleoporin

**DOI:** 10.1371/journal.pcbi.1000145

**Published:** 2008-08-08

**Authors:** V. V. Krishnan, Edmond Y. Lau, Justin Yamada, Daniel P. Denning, Samir S. Patel, Michael E. Colvin, Michael F. Rexach

**Affiliations:** 1 Department of Applied Science, University of California Davis, Davis, California, United States of America; 2 Department of Molecular, Cell and Developmental Biology, University of California Santa Cruz, Santa Cruz, California, United States of America; 3 Department of Chemistry, California State University Fresno, Fresno, California, United States of America; 4 Chemistry, Materials, Earth and Life Sciences Directorate, Lawrence Livermore National Laboratory, Livermore, California, United States of America; 5 Department of Biology, Massachusetts Institute of Technology, Cambridge, Massachusetts, United States of America; 6 Center for Computational Biology, School of Natural Sciences, University of California Merced, Merced, California, United States of America; University of California San Diego, United States of America

## Abstract

The nuclear pore complex (NPC) provides the sole aqueous conduit for macromolecular exchange between the nucleus and the cytoplasm of cells. Its diffusion conduit contains a size-selective gate formed by a family of NPC proteins that feature large, natively unfolded domains with phenylalanine–glycine repeats (FG domains). These domains of nucleoporins play key roles in establishing the NPC permeability barrier, but little is known about their dynamic structure. Here we used molecular modeling and biophysical techniques to characterize the dynamic ensemble of structures of a representative FG domain from the yeast nucleoporin Nup116. The results showed that its FG motifs function as *intra*molecular cohesion elements that impart order to the FG domain and compact its ensemble of structures into native premolten globular configurations. At the NPC, the FG motifs of nucleoporins may exert this cohesive effect *inter*molecularly as well as *intra*molecularly to form a malleable yet cohesive quaternary structure composed of highly flexible polypeptide chains. Dynamic shifts in the equilibrium or competition between *intra*- and *inter*molecular FG motif interactions could facilitate the rapid and reversible structural transitions at the NPC conduit needed to accommodate passing karyopherin–cargo complexes of various shapes and sizes while simultaneously maintaining a size-selective gate against protein diffusion.

## Author Summary

The nuclear pore complex is a molecular filter that gates macromolecular exchange between the cytoplasm and the nucleoplasm of cells. It contains a size-selective diffusion barrier at its center composed of proteins named FG nucleoporins. These nucleoporins feature large, structurally disordered domains that are highly decorated with phenylalanine–glycine (FG) sequence motifs. The dynamic structure of these disordered FG domains excludes them from classical structural biology analyses such as X-ray crystallography; thus, new approaches are needed to characterize their shape. Here computational and biophysical approaches were used to elucidate the ensemble of structures adopted by the FG domain of a nucleoporin. The analyses showed that the FG motifs function as intramolecular cohesion elements that compact the shape of the FG domain, forcing it to adopt loosely knit globular configurations that are constantly reconfiguring. Within the nuclear pore complex, dozens of these nucleoporin FG domains may stack as loosely knit globules forming a porous sieve that gates molecular diffusion by size exclusion.

## Introduction

**Figure 1:**
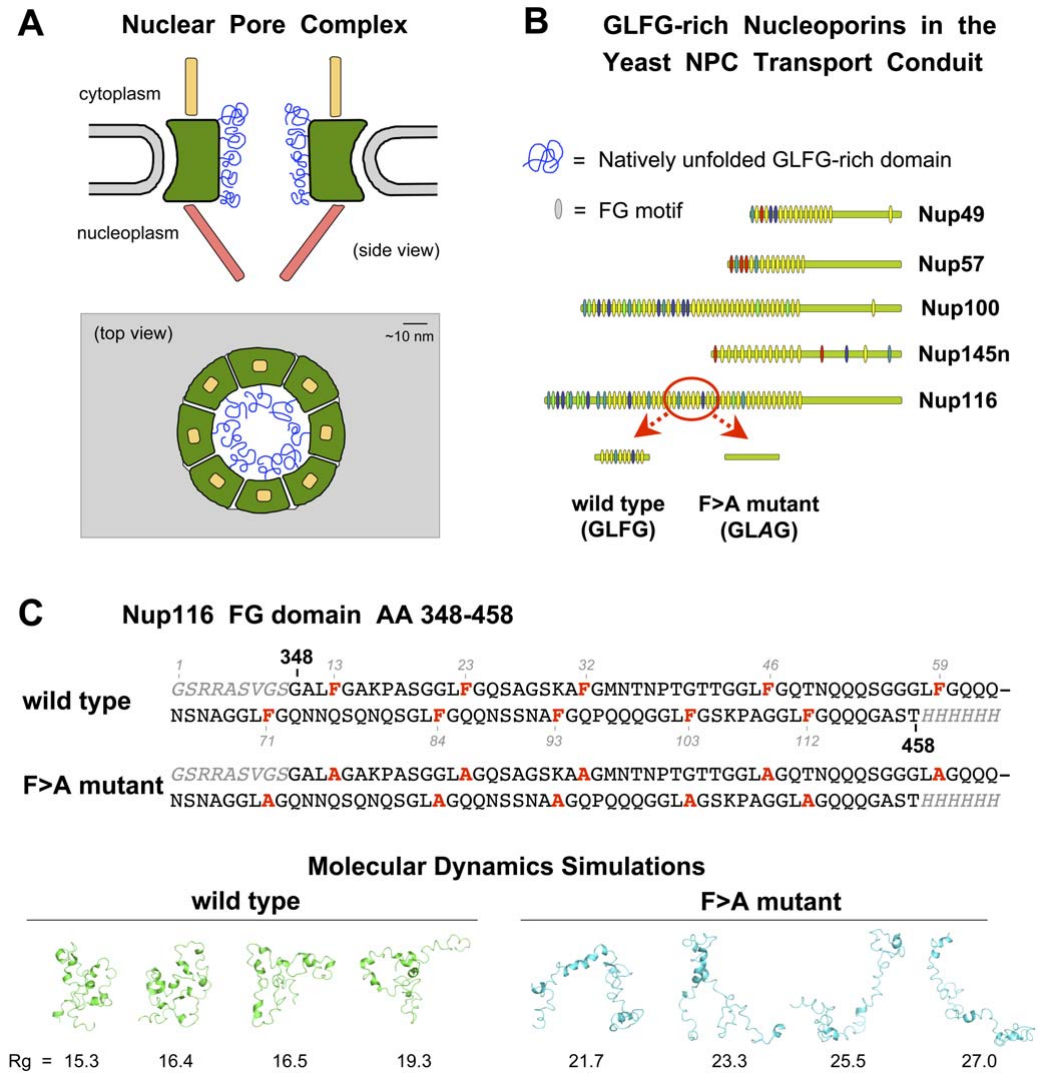
The yeast nuclear pore complex, some of its FG nucleoporins, and an ensemble of protein structures generated by MD simulations of a Nup116 FG domain. (A) A simplified diagram of the yeast nuclear pore complex (green), its cytoplasmic fibrils (yellow), its nuclear basket (red) and some of the GLFG-rich domains of nups that line the transport conduit (blue). The GLFG-rich domains are depicted as a doughnut-shaped array of laterally-cohesive, native pre-molten globules. Other FG domains are excluded for simplicity. The nuclear envelope is in gray. (B) GLFG-rich nucleoporins in the yeast NPC transport conduit. The vertical tick marks in the nups mark the location of each FG motif: GLFG motifs are in yellow, FxFG motifs in red, and other variants in different colors. The fragment of Nup116 (AA 348–458) indicated was selected as a representative FG domain for this study. The F>A mutant version lacks the phenylalanine residue in FG motifs, which were replaced by alanine. (C) The AA sequences for the wild-type and mutant Nup116 FG domains used for MD simulations. The phenylalanine (or alanine) in FG motifs is indicated in red. The AA sequences in gray are not part of Nup116; they are affinity-tags used in the purification of the FG domain. The numbers in the black bold font indicate the AA position in the Nup116 sequence. The numbers in gray indicate the AA position in the FG domain fragment analyzed. The protein structures shown are a representative subset of the twenty MD simulation structures generated for each FG domain at 350 K. The Rg values of the representative structures are within the average range for each FG domain (see Figure 2A).

The nuclear pore complex is a supramolecular protein structure in the nuclear envelope that controls nucleo-cytoplasmic traffic and communication (Figure 1A) [1]. A key NPC architectural feature is a poorly understood semi-permeable diffusion barrier at its center, which allows passive diffusion of particles less than 3–4 nm in diameter (or 30–40 kDa in mass for a folded protein) and opens to allow facilitated transport of larger particles up to 39 nm in diameter [2]. The NPC is composed of ∼30 proteins or nucleoporins (nups) that are present in multiple copies [3],[4]. Among these, a group that contains numerous phenylalanine-glycine repeats (FG nups) (a subset is shown in Figure 1B) line the transport conduit of the NPC (Figure 1A). These FG nups function as stepping-stones for karyopherin movement across the NPC [5],[6] and as structural elements of the NPC protein diffusion barrier [7],[8].

The three dimensional structure of *S. cerevisiae* FG nups is unusual because their 150–700 amino acid (AA) FG domains are natively unfolded [9] in their functional state [6]. Since there are ∼150 FG nups in each NPC [4], it is currently hypothesized that its transport conduit is lined and/or flanked by 150 natively unfolded FG domains. Together these FG domains constitute ∼12% of the total NPC mass or >6.5 MDa of its ∼55 MDa structure in yeast [10]. The FG domains of nups were initially hypothesized to function as repulsive entropic bristles that create a virtual gate at the NPC periphery [11],[12], and later as cohesive polypeptide chains that form a hydrogel at the NPC center [8],[13],[14]. More recently, an analysis of all nup FG domains in *S. cerevisiae* indicated that some FG domains (the GLFG-rich domains) bind to each other weakly via hydrophobic attractions between their FG motifs, whereas other FG domains (the FxFG-rich domains) do not form such cohesions [7]. Despite the fact that different subtypes of FG domains are defined by their content of FxFG, GLFG or SAFGxPSFG motifs, their ability to interact with each other (i.e., their cohesiveness) seems to correlate best with the AA composition of the sequences between FG motifs, rather than with the specific FG motif [7]. Hence, the human FG nups may also interact with each other, despite having only one GLFG-rich nup among its eleven members [3].

It is generally assumed that natively unfolded proteins have some preferred 3-D structures dictated by intra-molecular cohesion [15],[16]. Current evidence that the FG domains of nups have some structure is based on CD and FTIR spectroscopic analysis, which indicates that FG domains have anywhere from 5% to 20% α-helical and β-sheet content at any given moment [9], yet the locations of such structures in the protein are probably ever-changing. The conformational flexibility inherent to natively unfolded proteins and protein domains such as those in the FG nups, places them beyond the reach of classical structural biology tools such as X-ray crystallography and homology-based computational methods [17]–[20]. However, it is clear that these and other unfolded proteins participate in a wide range of key cell biological processes [21]–[23] and that their native plasticity bestows specific functional properties, such as rapid molecular interaction times and the ability to bind multiple proteins simultaneously [24]. In the case of nucleoporin FG domains, a key function is to bind multiple karyopherins [6] with very rapid interaction times [25]. Thus, in contrast to folded proteins, the structure of natively unfolded proteins must be described as a dynamic ensemble of interconverting conformers.

Since traditional experimental methods for elucidating protein structure cannot be used with natively-unfolded proteins, new approaches are needed to study and describe their dynamic ensemble of structures. In this emerging area of research, Jha et al [26],[27] have recently introduced a general statistical coil model, and Bernado et al [28],[29] have estimated the nuclear magnetic resonance (NMR) measured residual dipolar couplings (RDCs) [30] from dynamic simulations to characterize the ensemble-averaged conformations of α-synuclein. Also, Ollerenshaw et al [31] have applied a native-centric topological model to understand the essential folding/unfolding dynamics SH3 domains, and Pappu and co-workers have characterized poly-glutamines as a function of chain-length conformational sampling by molecular dynamics (MD) and Monte Carlo simulations [32]. Most of these computational investigations suggest the existence of a preferred ensemble of conformers for each protein, rather than suggesting pure random coils.

Here we conducted molecular dynamics simulations and biophysical measurements on a small FG domain from the yeast nucleoporin Nup116 (Q02630) to test the hypothesis that phenylalanines in its FG motifs function as *intra*molecular cohesion elements that impart structure. Apart from its cell biological significance, we chose this protein as a model system to investigate how a combination of molecular dynamics simulations and biophysical measurements can be used to characterize the ensemble of structures adopted by a natively unfolded protein, such as the FG domain of a nucleoporin.

## Results

In the analysis that follows we first used MD simulations to generate a statistical ensemble of coil conformations for a 111 AA region of the Nup116 FG domain containing ten FG motifs (wild-type), and of a mutant version thereof lacking the phenylalanines in the ten FG motifs (F>A mutant) (Figure 1B and 1C). The MD trajectories were then analyzed to evaluate the degree of secondary structure, the overall dimensions of the protein conformations, and the contribution of the FG motifs to the *intra*molecular cohesion of coils (i.e., compaction) in the dynamic ensemble of nup structures. The simulated FG domains were then expressed in bacteria, purified to homogeneity, and analyzed by NMR spectroscopy and sizing columns to quantify their average shape through measurements of diffusion coefficient and Stokes radii. Finally, mathematical and biophysical analyses were combined to estimate the tertiary structure that best describes the natively unfolded domain of the representative FG nucleoporin.

### Molecular Dynamics Simulations

Twenty independent MD simulations were performed at 300 K (25°C) on the wild-type (6 ns) and F>A mutant (5 ns) versions of a Nup116 FG domain (AA 348–458) starting from a fully-extended conformation. The goal of these simulations was to sample the conformational distribution of the proteins as close as possible to their native distribution in solution. As soon as the simulations started, within the first 100 ps, the extended FG domains collapsed into a more cohesive or compact ensemble of structures with small patches of unstable (see below) secondary structure. Since the wild-type and mutant FG domains are highly flexible and disordered, the resulting end-structures from each of the twenty simulations did not resemble one another as expected for natively unfolded proteins (see Figure 1C for representative examples). Despite the fact that the nup structures were ever-changing (see below), the ensemble of structures for each did “converge” to a similar size early in the simulation according to various metrics of size, which changed little in the last 3 ns. This was evidenced by a constant radius of gyration (Figure S1) and by statistical analyses that showed no significant change in the range of *R*_g_ values during the last 3 ns (data not shown).

To describe quantitatively the structural dynamics of the FG domains, we calculated the auto-correlation function of a vector of the 118 Φ and 118 Ψ angles along the peptide backbone of FG domain structures sampled every 1 ps from the MD trajectory. Figure S2 shows the autocorrelation functions with a 200 ps window from the final 3 ns of simulation of all twenty wild-type and F>A mutant FG domain simulations, along with the comparable auto-correlation function from the MD simulation of a control protein that is folded (fibroblast growth factor 1). For each of the replicate nup simulations, the correlation in the Φ–Ψ angles dropped from 1 to 0.738 (±0.031) or 0.741 (±0.023) in 1 ps for the wild-type or mutant FG domains, respectively, and then slowly decayed over 200 ps to 0.672 (±0.037) or 0.665 (±0.029), respectively. In contrast, for the control protein fibroblast growth factor 1, the autocorrelation function dropped to only 0.875 in 1 ps, and then to 0.864 over the 200 ps auto-correlation window. These results indicate that the AA chain backbone of wild-type and mutant FG domains is constantly changing structure and is highly dynamic in comparison to a folded protein.

### Structural Analysis of the Simulated FG Domains

The ensemble of structures for each of the twenty MD trajectories generated for the wild-type and mutant FG domains were sampled at 1 ps intervals during the final 3 ns of the simulations, yielding a total of 60,000 structures for each protein. The secondary structure content was then analyzed in detail to determine the fraction of time during the simulations that each AA residue spent as part of a “helical” structure (either an α-helix or a 3_10_-helix). In general, no significant difference in overall helical content between wild-type and mutant FG domains was observed. The alpha- and 3_10_- helical structures that did form ranged in size from 2–6 AA residues and did not persist for more than 35 ps on average (data not shown). The maximum duration of an α-helix and a 3_10_-helix was 97 and 699 ps, respectively (data not shown).

**Figure 2:**
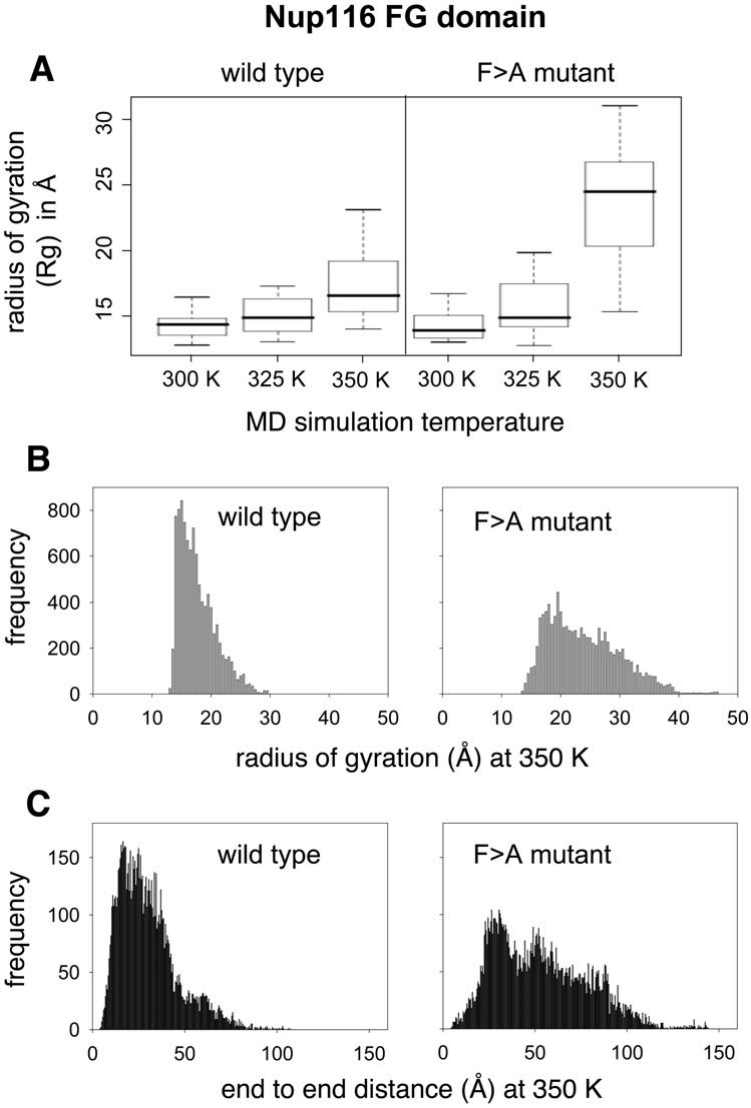
Molecular dimensions of the simulated FG domain structures. (A) Average radii of gyration of Nup116 FG domains simulated at different simulation temperatures. Box-plot of average radii of gyration (*R*_g_) in units of Angstroms calculated from twenty replicate 1 ns simulations at 300, 325, and 350 K for the wild-type and F>A mutant FG domains. (B) Histogram of radii of gyration (calculated using only the atoms in the peptide backbone) for the 10,000 FG domains structures sampled from the 350 K simulations. (C) Histogram of end-to-end distances (calculated from the terminal C and N atoms) for 10,000 FG domain structures obtained by sampling every 1 ps of the final 500 ps of each of the twenty replicate MD simulations at 350 K.

Using the same set of 60,000 structures, two measures of protein compactness were calculated: the radius of gyration (*R*_g_) and the end-to-end distance between terminal residues. The average (±1 standard deviation) end-to-end distance for the wild-type FG domain simulated at 300 K was 20.42 Å (±9.51), and for the mutant was 20.69 Å (±7.78) (data not shown). The predicted radius of gyration was 14.52 Å (±1.18) for the wild-type and 14.41 Å (±1.24) for the mutant FG domain (Figure 2A). The simulations sampled different regions of conformation space because significant run-to-run variations were observed in the probability distributions for each structural parameter. The similar *R*_g_ and end-to-end distance values obtained for the wild-type and mutant FG domains implied that both proteins occupy equivalent hydrodynamic volumes. However, this conclusion was at odds with two different quantitative measurements of the physical dimensions of purified FG domains (see below).

Interestingly, it has been reported that increasing MD simulation temperature can yield protein dimensions that more closely resemble those obtained by NMR protein conformation measurements [33],[34]. Indeed, when we extended the nup MD simulations for an additional 1 ns at 325 K (52°C) or at 350 K (77°C), a very different picture emerged (Figure 2A). At 325 K there was a slightly greater difference in the average radius of gyration between the wild-type (15.11±1.43 Å) and the mutant (15.76±2.58 Å) FG domains. Five of the twenty mutant simulations now had an average *R*_g_ greater than 18 Å, but all the wild-type simulations had an average *R*_g_ below 18 Å, indicating that the mutant FG domain is larger (Figure 2A). In addition, the average end-to-end distance for the wild-type FG domain was 20.84 Å (±10.75) compared to 24.26 Å (±13.16) for the mutant (data not shown). At 350 K, there was a much greater difference between their radii of gyration (*R*_g_). The average *R*_g_ was 17.40 Å (±3.11) for the wild-type FG domain and 23.68 Å (±6.05) for the mutant domain (Figure 2A and 2B). At 350 K, fifteen of the twenty mutant simulations had an average *R*_g_ greater than 20 Å, compared to only three for the wild-type simulations (data not shown). Consistently, the average end-to-end distance for the wild-type FG domain was 29.95 Å (±16.04) compared to 52.56 Å (±25.31) for the mutant (Figure 2C). The larger dimensions obtained for the wild-type and mutant FG domains at 325 K and 350 K compared to 300 K were likely due to thermal “melting” during the additional 1 ns of simulation. These data combined provide a first indication that the F>A mutant Nup116 FG domain is not as *intra*molecularly cohesive or compact as the wild-type version.

### *Intra*molecular Distances between FG Motifs in the Nup116 FG Domain

**Figure 3:**
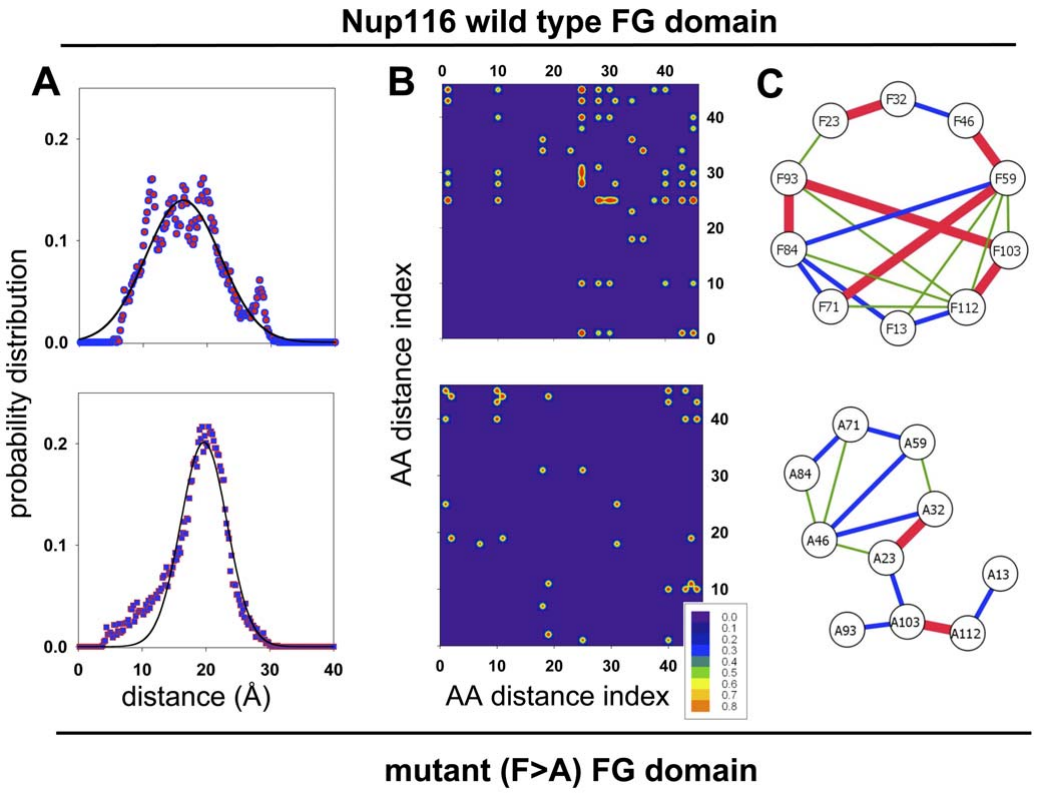
Interresidue distances in the simulated FG domains. (A) Plots of the probability distribution of inter-atomic distances in the wild-type and mutant FG domains. The distance distribution between the backbone β-carbons of phenylalanine (F) or alanine (A) residues in positions 84 and 93 (see Figure 1C) is shown as a representative example. The solid line in each plot corresponds to a Gaussian fit to the probability distribution. (B) Pearson squared correlation plots of the atomic distance between F–F or A–A pairs in the wild-type and mutant Nup116 FG domains. The numbers in the axes correspond to the various F–F or A–A pairs that result from all possible combinations (listed in Table S1). The correlation map shows how each of the pairs is related to the others. The insert depicts the contour level of the Pearson coefficient. (C) Schematic representations of Phe-to-Phe distances in the wild-type FG domain and Ala-to-Ala distances in the F>A mutant domain. The calculated average distance between the Phe or Ala residues in the pair-wise combinations is thickness and color coded. Thick red lines represent distances between 10 and 15 Å; medium blue lines represent distances between 15 and 20 Å; and thin green lines represent distances greater than 20 Å.

As a way of assessing the dynamic structure of the FG domain, particularly from the point of view of the FG motifs, we plotted the distances between the backbone β-carbons (Cβ) for the ten sites that correspond to the phenylalanine (Phe, F) or to the substitute alanine (Ala, A) residues in the various FG motifs. The distances used were from the MD simulations at 350 K, which yielded structures (Figure 1C) that better reproduced the dimensional difference between the wild-type and mutant FG domains measured by NMR analysis and in sieving columns (see below). The distance analysis yielded 45 F–F (or A–A) distances for each structure (Table S1). Probability distributions for each Cβ-to-Cβ distance were calculated and analyzed looking for significant differences between the wild-type and mutant FG domain configurations. To estimate the sharpness of the Cβ-to-Cβ distance distributions, the number of 1 Å wide bins that had greater than 10% of the probability distribution was counted; no bin had more that 20% of the probability distribution. This metric was calculated for all 45 Phe–Phe Cβ-to-Cβ distances in all twenty replicates of the FG domain simulations. In stable tertiary structures, these distances occur as one sharp-peak distribution around the equilibrium inter-residue distance; in a fully random ensemble of structures, they occur as a very broad distribution; and in semi-structured proteins, they occur as one or more intermediate-width distributions. Figure 3A shows two representative examples of the probability distributions obtained from the MD trajectories at 350 K; the values shown correspond to the distribution of distances between phenylalanine F84 and F93 in the wild-type FG domain (Figure 1C) or alanine A84 and A93 in the F>A mutant domain. Overall, for the wild-type FG domain simulations, the majority of the simulations analyzed had more than three peaks; by comparison, only a minority of the F>A mutant simulations analyzed exhibited a similar level of sharpness in the distance distributions (data not shown). These results provide tentative evidence that the wild-type FG domain is more structured than the mutant. Repeating this analysis to include only peaks with the distance distributions of <15 Å yielded a very similar result (data not shown).

To permit comparisons of the average inter-residue distances, the probability distributions obtained for the Cβ-to-Cβ distances were fit to a single Gaussian distribution even though in some cases there were multiple distinct peaks (Figure 3A). This was only a rough approximation to the observed probability distribution, but the assumption was justified in the context that these ensemble of structures were to be used (see below). After all, these structures are rapidly inter-converting and the width of the Gaussian is broad enough to accommodate all of the major peaks in the distribution. For example, in the case of the F84–F93 distance distribution, a Gaussian centered at 16.2 Å with a width of 11 Å covered both peaks at 10 and 20 Å (Figure 3A).

Probability distributions of all inter-residue distances obtained from the MD simulations were subjected to clustering using the Pearson squared correlation. This was done to determine how any two of the distributions sampled in regular intervals of the MD simulations are correlated with each other. The correlation coefficient does not depend on the specific measurement units used because other correlation coefficients, such as Euclidian distance metric, yielded similar clustering effects (data not shown). Figure 3B shows the matrix intensity plots of the correlations for the wild-type and mutant FG domains. The indices of the matrix correspond to the various Phe–Phe pairs (or Ala–Ala pairs) listed in Table S1. Indices 1 through 9 correspond to the distance from the first Phe (at position 13; see Figure 1C) to the other 9 Phe (positions 23 through 113), while indices 10–18 correspond to similar distances from the second Phe (at position 23) to the other eight Phe's (positions 32 through 113) and so on. Altogether, the clustering analysis showed that there is a stronger correlation between the various Phe–Phe distributions in the ensemble of wild-type FG domain structures than between the various Ala–Ala distributions in the ensemble of mutant FG domain structures. This indicated that the wild-type FG domain is generally more ordered than the mutant.

To obtain a broader view of the dynamical correlation between inter-residue distances in the FG domains, the Pearson correlation coefficient between all 990 distinct interresidue distances in all 20 simulation replicates of each FG domain were calculated, yielding 19,800 correlation coefficients. In this case, a value of 1.0 would indicate a perfect linear correlation between two inter-residue distances (as one inter-residue distance grew larger, the other would grow by a proportionate amount); a value of 0.0 would indicate no correlation between a distance pair; and a value of −1.0 would indicate perfect anticorrelation. Figure S3 shows back-to-back histograms of the resulting correlation coefficients. For the wild-type FG domain, 9.0% of the observed correlation coefficients had values above 0.7 versus 7.1% in the F>A mutant. This demonstrated that the ensemble of wild-type FG domain structures shows a bias towards higher correlation coefficients between inter-residue distances than the F>A mutant domain, indicating more structural coherence in the wild-type FG domain than in the mutant.

To better describe the relationship between FG motifs in the FG domain, the distances between F–F pairs (or substitute A–A pairs) were also categorized into groups representing distances of 10–15, 15–20, or >20 Å. These are shown in Figure 3C as thick red, medium blue, or thin green lines, respectively. A list of all distances for both proteins is given in Table S2. Among the F–F distances in the wild-type FG domain, seven F–F pairs are less than 15 Å apart (red text in Table S2 and red-thick lines in Figure 3C). In contrast, the F>A mutant FG domain had only two A–A pairs with such short distances. In the wild-type FG domain, four F–F pairs were in the range of 15–20 Å apart (blue), while seven F–F pairs were farther than 20 Å (green). In the F>A mutant, there were eight A–A pairs with distances in the mid-range (15–20 Å), and three pairs showing distances greater than 20 Å. These results demonstrate that the *intra*molecular distances between FG motifs in the wild-type and mutant FG domains are quantifiably different from each other. There was a tendency for FG motifs in the wild-type FG domain to be proximal to each other (i.e., to cluster), which was absent in the mutant. This conclusion is consistent with the hypothesis that the FG motifs in the wild-type Nup116 FG domain interact *intra*-molecularly in a manner similar to what has been observed for *inter*molecular interactions between this Nup116 FG domain and other FG domains of nups [7].

### Self-Diffusion Coefficient Measurements of Nup116 FG Domains by NMR

**Figure 4:**
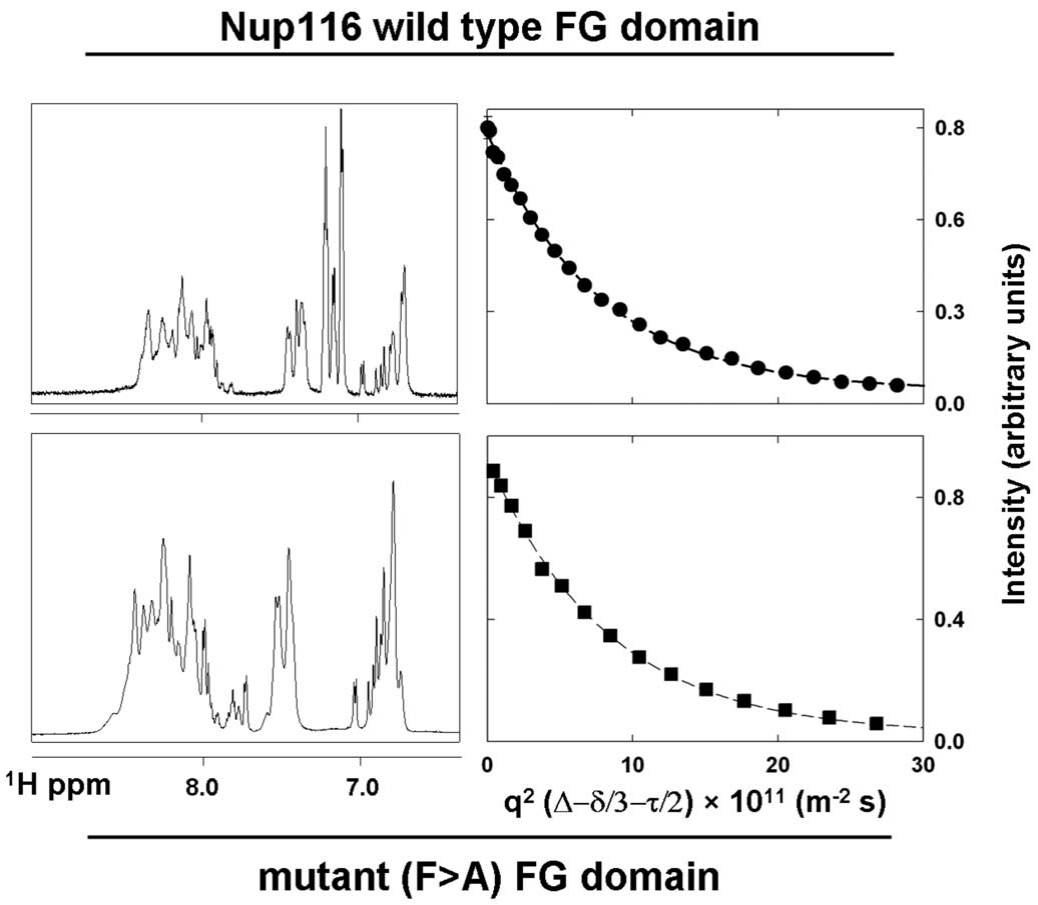
Analysis of Nup116 FG domains by NMR. *Left panels:* Aromatic and amide region of the water-gate residual water suppressed one-dimensional NMR spectra for the purified FG domains. Tall peaks in the spectrum between 7.1 and 7.3 ppm arise from the Phe residues in the wild-type domain, which are absent in the F>A mutant. The sensitivity of the mutant FG domain spectrum is lower due to a lower protein concentration than the wild-type FG domain. *Right panels:* Plot of the self-diffusion coefficient measurements performed using BPP-SED for the wild-type and mutant FG domains. Circles depict the experimental points and squares, and lines correspond to the fit to the diffusion data. The error bars are smaller than the size of the symbols.

The structural predictions made by the in silico modeling prompted us to seek physical evidence that the phenylalanine residues in FG motifs function as structural cohesion elements that form putative *intra*-molecular interactions within the Nup116 FG domain. In principle, a change in the dynamic ensemble of FG domain structures resulting from the substitution of all Phe's to Ala's could be detected by NMR. A less-ordered mutant FG domain would exhibit a slower diffusion coefficient. The wild-type and F>A mutant versions of the Nup116 FG domain were purified to `homogeneity and subjected to NMR analysis. Plots of the one-dimensional ^1^H NMR spectra are shown in Figure 4 (left panels). It was anticipated that the hydration of the FG domains would be significantly different from that of ordered, globular proteins [35],[36] due to the lack of stable folded structures in the FG domains [9]. When presaturation of the water was used there was a significant reduction in the intensity of the amide region of the nup spectrum due to fast exchange with the solvent protons [37],[38]. This observation, combined with the narrow chemical shift dispersion of the amide resonances (7.9–8.5 ppm) in both nup spectra, was a clear indication that both FG domains are natively unfolded and highly dynamic. NMR experiments conducted at lower temperatures (5 and 10°C, compared to 25°C) gave similar results, but offered no significant improvement in the spectral dispersion (data not shown).

Experimental self-diffusion measurements (intensity vs. product of the area of gradient pulse strength and the diffusion length) of the FG domains and the corresponding exponential fits are also shown in Figure 4 (right panels). The data yielded self-diffusion coefficients (*D*_s_^expt^) values of 13.17 (±0.26) and 12.18 (±0.12)×10^−11^ m^2^ s^−1^ for the wild-type and mutant FG domains, respectively. This indicated slower diffusion for the less-ordered mutant FG domain. Despite the mass of the F>A mutant domain being smaller (11.9 kDa) than wild-type (12.6 kDa) (due to the replacement of 10 Phe for Ala) the diffusion constant of the mutant was smaller on average, suggesting that its effective hydrodynamic volume is larger. As expected for unfolded proteins [39], the diffusion of the wild-type and mutant FG domains was significantly slower than a folded protein of higher molecular weight [39],[40], indicating that the wild-type and mutant FG domains have unfolded structures that sample a relatively large conformational space.

### Characterization of the Nup116 FG Domain in Sizing Columns

To further characterize the hydrodynamic properties of the wild-type and mutant Nup116 FG domains, each was analyzed by FPLC in a sieving column to determine its Stokes radius. The expectation was that the less ordered mutant FG domain would occupy more hydrodynamic space and would elute faster from the sizing column. Purified wild-type and mutant versions of the Nup116 FG domain were subjected to size-fractionation through an FPLC Superdex 75 column and their elution profiles were compared to that of commonly-used size standards, such as carbonic anhydrase (29 kDa, *R*_s_ = 23.5Å), ovalbumin (45 kDa, *R*_s_ = 29.8Å), and BSA (68 kDa, *R*_s_ = 35.6Å). The Stokes radius for the wild-type FG domain was measured at 25.2 (±0.6) Å (Table 1), which is larger than carbonic anhydrase despite the FG domain having less than one-half the mass. This highlighted the fact that the FG domain is natively unfolded. The F>A mutant domain eluted faster from the sieving column and migrated as a particle with a Stokes radius equivalent to 27.1 (±0.6) Å, which is larger than the wild-type FG domain despite the mutant having less mass (Table 1). This apparent loss of compaction for the mutant FG domain compared to the wild-type (a ∼20% change in hydrodynamic volume) was consistent with its slower NMR diffusion coefficient (Figure 4) and with the computationally-predicted difference in hydrodynamic dimensions between them at 350 K (Figure 2). The observed loss of intra-molecular cohesion in the mutant FG domain supported our hypothesis that FG motifs within the natively-unfolded FG domain of Nup116 interact *intra*molecularly via phenylalanines that cluster through hydrophobic attractions. In essence, the hydrophobic interactions between FG motifs likely bias the arrangement of coils within an FG domain to form an ensemble of dynamic non-random tertiary structures with a quantifiable level of *intra*molecular cohesion.

**Table 1:** Hydrodynamic dimensions of the purified Nup116 FG domains.

		Estimated Stokes radius from MD simulations at 350 K	Measured Stokes radius		Predicted Stokes radius (*R*_s_ in Å) based on mass^a^					
FG domain	AA's	*R*_h_ = *R*_s_ (Å)	*R*_s_ (Å)	MW (Da)	Folded	Molten globule	Premolten globule	Coil	Extended coil in urea	Extended coil in GnHCl
Nup116	348–458	23.1±4.1^b^	25.2±0.6^b^	12,632^b^	16.9^b^	20.3*	**26.2** b	28.6*	29.9^b^	31.2^b^
Nup116 F>A	348–458	29.6±5.4^b^	27.1±0.6^b^	11,861^b^	16.6^b^	19.9*	25.6^b^	**27.8** b	28.9^b^	30.1^b^

Boldface highlights the best-match between measured Stokes radius and predicted Stokes radius based on mass.

a Predicted Stokes radii based on mass using Uversky's equations [41].

b Includes a 9 AA tag at the N-terminus (858 Da) and a 6 AA His-tag (841 Da) at the C-terminus in addition to the nup sequence (see Figure 1C).

### The Measured Hydrodynamic Volume of the Nup116 FG Domain Predicts a Native Premolten Globular Structure

The hydrodynamic volume or Stokes radius of a protein in different structural configurations (e.g., a folded globule, a molten globule, a premolten globule, a coil, an extended coil) can be estimated from its mass using mathematical equations [41]. These equations were derived from the analysis of large data sets containing experimentally-determined hydrodynamic values for proteins in those structural configurations. Here, using the mass of the wild-type and mutant Nup116 FG domains, we calculated their hypothetical Stokes radius in each structural configuration and compared these predicted values to our experimentally-measured Stokes radii values (Table 1). The goal was to identify the structural configuration of each FG domain that best matched the biophysical measurement obtained for its hydrodynamic volume. For the wild-type FG domain, a predicted native pre-molten globule structure matched best its measured Stokes radius, and for the F>A mutant, a predicted native coil structure was the best match (gray boxes, Table 1). These results support the hypothesis that GLFG motifs in nucleoporins function as intra-molecular cohesion elements, because their absence caused a loss of compaction in the Nup116 FG domain, shifting its dynamic ensemble of structures from native premolten globular configurations to native coil configurations.

## Discussion

We have used a combined computational and biophysical approach to characterize the dynamic ensemble of structures adopted by a natively unfolded or intrinsically unstructured protein. Specifically, we characterized the ensemble of conformations adopted by a fragment of the FG domain of the *S. cerevisiae* nucleoporin Nup116 (AA 348–458) and of a mutant version thereof (F>A) lacking the phenylalanines in its predominantly GLFG motifs (Figure 1). Both FG domains were found to be highly dynamic and disordered, yet contained quantifiable structural differences between them. The MD simulations predicted a more cohesive and/or compact ensemble of structures for the wild-type FG domain compared to the F>A mutant based on the average radius of gyration and end-to-end distances (Figure 2). This structural prediction was supported by the inter-phenylalanine or inter-alanine distance analysis (i.e., the distance between wild-type or mutant FG motifs, respectively) (Figure 3C), which indicated shorter distances between the FG motifs in the wild-type domain; and by the Pearson correlations of F–F (or A–A) pair distances in the FG domains (Figure 3B), which indicated that the Nup116 FG domain has increased probability of sampling geometries that are more ordered when the phenylalanines in the FG motifs are present. The structural predictions made by the MD simulations were confirmed by direct physical examination of purified FG domains, through NMR-based measurement of their hydrodynamic properties (Figure 4) and by measurement of their hydrodynamic radii in sieving columns (Table 1). In all of the analyses, the wild-type FG domain was found to be more compact than the mutant domain. Unlike the simulations at 350 K, the lower temperature simulations (e.g., at 300 K) did not reproduce this difference in hydrodynamic volumes. Hence, the MD simulations at the higher temperature (350 K) for this class of natively-unfolded proteins may reproduce more accurately their physical properties in solution. As a caveat, the magnitude of the size difference between the nups simulated at 350 K is larger than the magnitude of the difference in their physical dimensions as measured in the sizing columns (Table 1). Notwithstanding, the simulation values matched, within the experimental error of the simulations (s.d. ±18%), the measured Stokes radii for the purified FG domains. Since only 20 single-molecule simulations were used to predict the dimensions of the FG domains, whereas ∼35 trillion molecules were used to accurately measure their average dimension in the sieving columns (s.d. ±2%), it seems likely that a greater number of simulations for greater time-periods could increase the congruency between simulated and measured values.

The mass and physical dimensions of the Nup116 FG domain fragment analyzed here (AA 348–458; MW = 12.6 kDa; *R*_s_ = 25.2±0.6), together with the scaling relations developed by Uversky's group [41], led us to conclude that this Nup116 FG domain fragment is best described as a dynamic ensemble of native pre-molten globular structures (Table 1). This structural information can in turn be used to predict the physical dimensions of the full-length Nup116 FG domain (AA 1–960; see [42]) based on its mass and assuming that it also adopts native premolten globular structures. Using the scaling relations, which convert protein mass to physical dimensions in any of a number of structural configurations [41], we estimated that the entire Nup116 FG domain would occupy a hydrodynamic volume equivalent to a 12-nm-diameter sphere (Table S3). For comparison, its volume would be equivalent to a 16-nm-diameter sphere if it were to adopt less compact native-coil configurations; or to a 19-nm-diameter sphere if it were to adopt extended-coil configurations; or to a 7-nm-diameter sphere if it adopted a tightly folded configuration (data not shown). Likewise, size estimations can be done for other full-length nucleoporin FG domains that have similar AA composition and FG motif type as Nup116 (e.g., the GLFG nup subfamily shown in Figure 1B) [42]. Such analysis predicts that their FG domain dimensions would be equivalent to spheres with diameters of 7, 7, 11, and 7 nm for Nup49 (AA 1–251) (Q02199), Nup57 (AA 1–255) (P48837), Nup100 (AA 1–800) (Q02629), and Nup145n (1–216) (P49687), respectively, assuming native premolten globular configurations for each case (Table S3). These predicted dimensions for the FG domains are generally consistent with the 16–46% larger dimensions reported for the full-length FG nups containing the FG domain, the folded NPC anchoring domain and a Protein A tag (Table S3) [43]. Interestingly, all of these FG domains including the Nup116 FG domain appear to be large enough to butt against each other locally within the NPC (at least within a single spoke and probably between adjacent spokes) given their close anchoring at the NPC (see paragraph below) [43],[44], yet appear to be too small to span *across* the NPC transport conduit from their tether sites within the NPC scaffold (∼19 to 32 nm away from the conduit center; Table S3) to the space occupied by the FG domains of nups anchored at the opposite side (see Figure 1A and Figure S5A). This is because the NPC transport conduit has an estimated radius of 19 nm [2],[43],[44], which is significantly larger than the estimated diameter for these FG domains (∼7–12 nm). Notwithstanding, large fluctuations in the dimensions of FG domains, which are intrinsic to natively unfolded structures, or steric hindrance effects caused by the spatial confinement between closely-anchored FG domains [12],[45] could cause the FG domains to extend further out into the transport conduit (Figure S5B). The cohesive properties between FG domains within the conduit [7],[14] (also see below), or the cross-linking action of karyopherins within the conduit (i.e., karyopherins appear to bind multiple FG motifs in different FG domains simultaneously) [45]–[47] could transiently stabilize some of the extended FG domain conformations (Figure S5B and S5C, respectively) [14].

The evidence presented here suggests that the FG motifs in Nup116 function as structural, *intra*molecular cohesion elements that bias the arrangement of coils within the FG domain and condense its dynamic ensemble of structures into more cohesive, less disordered states. In the case of the Nup116 FG domain examined, its FG motifs are responsible for shifting its ensemble of structures from native-coil configurations (as seen for the mutant) to native pre-molten globular configurations (Table 1 and Figure 1C). In principle, all types of FG motifs (GLFG, FxFG, SAFG, PSFG, etc.) [42] could exert cohesion through hydrophobic pairing, stacking, zippering, or otherwise clustering of the aromatic ring of phenylalanine side chains through energetically favorable aromatic edge-to-face interactions, as opposed to less favorable face-to-face (π–π) interactions [48]. Interestingly, a report by Dhe-Paganon et al., defined a “phenylalanine zipper” motif within the hydrophobic core of APS, which is critical for APS dimerization [49]. There, the aromatic side chains of ten phenylalanine residues are uniquely stacked to form a zipper that is stabilized by helical secondary structures in the protein backbone. Although FG domains do not appear to have stable secondary structures, residues surrounding the FG motif, such as the leucine residue of GLFG motifs or the second phenylalanine residue in FxFG motifs, could enhance the hydrophobic clustering effect by increasing the local hydrophobicity of the Phe residue in the FG motif and/ or by influencing the orientation of its Phe ring. A two dimensional representation of the Nup116 FG domain AA sequences in a hydrophobic cluster analysis (HCA) [50],[51] illustrates its hydrophobic “LF” patches very well (Figure S4). Although HCA is most commonly used in determining hydrophobic clusters in helical patterns [31], it is also informative in the absence of a structural fold because it allows the identification of hydrophobic features between nearby AAs. The HCA analysis highlighted LF patches and MFMF-connections in the wild-type Nup116 FG domain, which were missing in the F>A mutant domain. This implied that the F>A mutant FG domain is less compact because it does not have hydrophobic patches and connections to make intra-molecular interactions.

Our finding that the FG motifs can function as *intra*molecular cohesion elements has important implications for the general architecture and function of the NPC, especially if the ability of these FG motifs to mediate *intra*molecular cohesion of coils functionally mimics their demonstrated ability to mediate *inter*molecular cohesion between FG domains [7]. Indeed, the representative FG domain of Nup116 analyzed here and the FG domains of other (but not all) FG nups (Nup49, Nup57, Nup100, nNup145, and Nup42) engage in homotypic and heterotypic interactions with each other in vitro and in vivo via FG motifs [7]. By analogy to the Nup116 FG domain, this group of cohesive FG domains may also exhibit *intra*molecular cohesion of their own FG motifs. At the NPC, such *intra-* and *inter*molecular FG motif interactions could be in competition with each other, possibly causing the FG domains to fluctuate between monomeric and polymeric states (Figure S5B). Alternatively, such interactions could be in a dynamic equilibrium with each other to form a metastable quaternary structure (Figure S5A). According to the two-gate and the hydrogel models of NPC architecture, the FG motifs of nups within the NPC conduit engage in *inter*molecular cohesions with each other to form a highly flexible network of cohesive polypeptide chains, which forms a size-selective sieve or gate [7]–[9],[13],[14]. The cohesiveness of such network(s) is presumably maintained by the weak but numerous interactions between FG motifs [52]. However, if the *intra-* and *inter*molecular interactions between FG motifs were in competition with each other at the NPC, then *intra*molecular cohesions could effectively prevent the FG domains from forming a network altogether by causing them to “fold back” on themselves (i.e., an autoinhibitory mechanism).

What type of FG motif interaction dominates at the NPC, either *intra*molecular or *inter*molecular, is indeed an important question whose answer may rely largely on four parameters: the distance between FG domain anchor sites at the NPC, the volume of space occupied by each FG domain, the space available at the NPC for each FG domain, and the steric hindrance effect between neighboring FG domains [12],[45],[53]. As discussed above, the estimated dimensions for the “cohesive” GLFG-rich domains of yeast nups (7–12 nm diameter spheres) combined with the close proximity between their anchor points within each spoke (most are ≤5 nm apart from others anchored adjacently and ≤10 nm from others anchored above or below in the *z*-axis; see Table S3) implies that at least within a spoke and probably between adjacent spokes the FG domains of these nups butt against each other to occupy overlapping space [43],[44]. This close positioning could allow or even promote the formation of a supra-molecular quaternary structure of cohesive FG domains at the NPC through a multitude of *inter-*molecular FG motif interactions. This structural assembly could take the form of a meshwork of intertwined polypeptide chains [14],[52], or alternatively, based on the data presented here, the assembly could take the form of a doughnut-shaped array of laterally-cohesive, native pre-molten globules (as depicted in Figure 1A and Figure S5A). Most importantly, local reversible shifts in the equilibrium between *intra*- and *inter*molecular FG motif interactions could facilitate the fast structural changes in the NPC permeability barrier, which are presumably coupled to the passage of karyopherin-cargo complexes of different shapes and sizes during transit across the NPC (Figure S5C). As karyopherin–cargo complexes disrupt (either by mass action or by direct interaction with the nup FG domain) *inter*molecular FG motif interactions during transit (as predicted for all FG nups in the hydrogel model, or for a discrete subset of nups in the two-gate model) [7],[8],[14], the FG motifs liberated as a result would become available to form *intra*molecular interactions. This could cause the FG domains to fold back on themselves (i.e., to compact), effectively opening the permeability barrier by suddenly occupying less space.

It remains to be determined whether other types of nup FG domains, which do not display *inter*molecular cohesions with each other via FG motifs [7], can nevertheless form *intra*molecular cohesions of their own FG motifs to adopt compact configurations. According to the “virtual gate” [11], the “oily spaghetti” [54] and the “two gate” [7],[55] models of FG domain architecture, these FG domains would exist as highly-extended polypeptide chains, as observed for the *Xenopus* Nup153 FG domain [12]. Interestingly, in the case of Nup153, its extended FG domain appears to compact upon binding a karyopherin [45]. Clearly, a more detailed structural characterization of the various FG domains as they interact with karyopherins and each other is needed to fully understand the dynamic and highly flexible structure of the NPC transport conduit.

## Materials and Methods

### Classical Molecular Dynamics Simulation

MD simulations of individual FG domains were started from a fully extended backbone structure (i.e., with the Φ and Ψ angles set to 180° for all residues except for the three proline residues, which put a 60° bend in the sequence). A different random number seed was chosen for each of the different simulations to randomize the initial atom velocities. Twenty separate simulations were run for either 6 ns (wild-type) or 5 ns (mutant) each using different initial atomic velocities and analyzed at 1 ps intervals. The wild-type fragment required an additional nanosecond of dynamics to have its radius of gyration converge. All MD simulations were performed with AMBER [56]–[58] using implicit solvent models. Each molecule was simulated in the presence of a Generalized Born/Surface Area (GB/SA) implicit solvent model [59] that calculates an effective solvation energy as an empirical parameter multiplied by the exposed surface area of different atom types. Each molecule was simulated using the GB/SA implicit solvent implementation in Amber versions 7 and 8. Each system is energy-minimized using 100 cycles of conjugate gradients. Constant-temperature molecular dynamics at 300 K with a coupling constant of 2.0 ps was performed on the minimized systems using the standard partial charges for the Amber force field and Bondi radii for the atoms. Bonds containing hydrogens were constrained using SHAKE and a time step of 2 fs was used in all simulations. A cutoff of 250 Å was used for the electrostatic interactions, which for this system is equivalent to infinity. The salt concentration (Debye-Huckel screening) was set at 0.15 M. *Secondary structure analysis:* For the final 3 ns of each simulation, the structure was analyzed every 1 ps using a standard program for identifying secondary structure from atomic coordinates (Define Secondary Structure of Proteins; DSSP) [60]. *Radius of gyration and end-to-end distance analyses:* For the final 3 ns of each simulation, radii of gyration and the end-to-end distances between terminal residues were calculated using the program CARNAL and ptraj, distributed with AMBER 7 [57],[58]. *High temperature molecular dynamics simulations:* Molecular dynamics were performed at elevated temperatures for each of the 20 wild-type and mutant FG domain simulations. The GB/SA simulations were all restarted after 5 ns coupled to a heat bath at 325 or 350 K with all other parameters of the simulation kept the same. The simulations were run for 1 ns, and the final 500 ps were used for analysis.

### Autocorrelation Functions

To determine the degree of dynamical change in the ensemble of FG domain structures, the autocorrelation function was derived for a vector (total vector length = 236) composed of the 118 Φ and 118 Ψ angles (in the 111 AA nucleoporin sequence with a 9 AA N-terminal tag; see Figure 1) along the peptide backbone of structures sampled every 1 ps from the MD trajectory. The autocorrelation function was computed as the dot-product of successive Φ–Ψ vectors using every 1 ps step as a new time origin and calculating the correlation function out to 200 ps. Several different time windows were used in the autocorrelation calculation, but all gave the same result. For comparison purposes, the same autocorrelation function was calculated for the first 118 Φ–Ψ angles (out of 130) for an MD trajectory of a well-folded protein (fibroblast growth factor 1; PDB ID = 1AXM).

### Calculation of Probability Distributions

The MD trajectories of 45 F–F distances between the Cβ atoms were analyzed to calculate the probability distributions. Systematically, each of the F–F distance was interrogated and all of the distances were binned (1 Å bins from 0 to 60 Å) to form a histogram of distance distributions. Probability distributions were calculated for each of the twenty simulations independently, and the values obtained were averaged at the end. A similar procedure was adopted for the mutant FG domain where the distance between the Cβ atoms of the Ala residue was used. Final probability distributions were used without any normalization.

### *Intra*molecular Distance Constraints

As a first approximation, the probability distributions were fit to a Gaussian distribution (probability versus distance). This is a conservative approach and is expected to be valid considering the number of structures generated (60,000) during the molecular dynamics simulations and in the absence of any constraints. The center of the Gaussian is considered as the mean distance between the F–F (or A–A), while the width at half-maximum is used as the allowed variation in the constraint. *Clustering analysis:* The correlation between different F–F probability distribution reflects the degree to which these variables (F–F distances) are related. The most common measure of correlation is the Pearson Product Moment Correlation (http://www.r-project.org/) and reflects the degree of linear relationship between the two variables. In order to determine whether probability profiles of the F–F interaction correlate, a similarity matrix with a Pearson square metric was calculated. The correlation was used to indicate the presence (or absence) of relationship between various F–F interactions.

### Synthesis, Expression, and Purification of FG Domains

The coding sequence for the representative 111 AA Nup116 FG domain was amplified from genomic *S. cerevisiae* DNA using PCR and was cloned into the vector pGEX-2TK in frame with the coding sequence for glutathione S-transferase (GST) at the 5′ end, and in frame with the coding sequence for six contiguous histidines at the 3′ end. Site directed mutagenesis was then used to alter the coding sequence for the mutant F>A FG domain. The correct coding sequences were confirmed by DNA sequence analysis. The FG domains were expressed in a *E. coli* BL21+ strain as fusion proteins with GST (glutathione S transferase) at the N-terminus and a HIS tag (six contiguous histidine residues) at the C-terminus. Glutathione coated Sepharose beads were then used to isolate each GST-FG domain fusion from crude bacterial cell extracts. The isolated FG domains were eluted from the beads by specific thrombin proteolysis of the GST tag. Nickel-coated beads were then used to capture and isolate the FG domain through its C-terminal His-tag, and the captured proteins were eluted from the beads using imidazole. Finally, the eluates were concentrated in a Centricon 3 unit and were size fractionated in an FPLC Superdex 200 sizing column that was equilibrated in 50 mM NaH_2_PO_4_, pH of 6.5 for the NMR analysis, or in an FPLC Superdex 75 column equilibrated in 20 mM Hepes, pH 6.8, 150 mM KOAc, 2 mM Mg(OA)_2_ for determination of Stokes radii.

### Determination of Stokes Radii

Tandem-affinity purified wild-type and F>A mutant Nup116 FG domains were subjected to size-fractionation through an analytical-scale FPLC Superdex 75 column. FG domains (100 µl of 7.5 mg/ml) were injected at a flow rate of 0.5 ml/min at 4°C into a column that was preequilibrated in 20 mM Hepes pH 6.8, 150 mM KOAc, 2 mM Mg(OAc)_2_, and 0.5 ml fractions were collected. The FG domain elution profiles were monitored by UV absorbance at 280 nm and by SDS-PAGE analysis of the eluates. The nup elution profiles were compared to those of carbonic anhydrase (29 kDa, *R*_s_ = 23.5 Å), ovalbumin (45 kDa, *R*_s_ = 29.8 Å), and BSA (68 kDa, *R*_s_ = 35.6 Å), which served as molecular size standards. The elution volume of the standards was plotted in relation to their Stokes radii, allowing for estimation of the FG domain Stokes radii from the resulting linear regression formula.

### NMR Experiments

NMR experiments were performed on tandem-affinity purified FG domains dissolved in 50 mM NaH_2_PO_4_, pH 6.5. Final protein concentrations were ∼0.5 mM for both wild-type and mutant FG domains. NMR experiments were performed in a Varian INOVA 600 MHz spectrometer equipped with a 5 mm probe with a single-axis (along Z) shielded magnetic field gradients. One dimensional ^1^H NMR experiments were obtained using the water suppression scheme 1-3-3-1 Water-gate [61]. Self-diffusion coefficient measurements were obtained using a BPP-SED (bipolar-gradient pulse pair selective echo dephasing) sequence [62].

### Hydrodynamic Calculations

Translational diffusion tensor values were calculated based on the beads-model approximation of García de la Torre and Bloomfield [63]. This method has been used successfully to calculate translational as well as rotational diffusion tensors of proteins [40],[64]. All atoms were considered as beads of equal size (σ = 5.1 Å). The overall isotropic translational self-diffusion coefficient was calculated by taking the average of the principal values of the diffusion tensor.

### Calculating Hydrodynamic Radius from the Radius of Gyration for the Simulated FG Domains

The hydrodynamic radius (*R*_h_) for the wild-type and mutant Nup116 FG domains was calculated from the radius of gyration (R_g_) values obtained from the simulations using the scaling relationship given in [39]. For native proteins, the scaling relationship is *R*_h_ = *R*_g_/0.77, and for proteins in strong denaturing conditions, the scaling relationship is *R*_h_ = *R*_g_/1.06. For the wild-type and mutant Nup116 FG domains simulated at 300 and 325 K, the hydrodynamics radius was obtained by *R*_h_ = *R*_g_/0.77. In the 350 K simulations, some of the protein conformations were highly extended (as in denaturing conditions) and a single scaling value was not appropriate. In this case, if the *R*_g_ for a structure was less than 30.7 Å for wild-type and 29.6 Å for the mutant, it was scaled by 1/0.77; if the *R*_g_ was greater, the value was scaled by 1/1.06. The *R*_g_ cutoff values of 30.7 Å (wild-type) and 29.6 Å (mutant) were obtained by using Uversky's relationship: *R*_h_ (8 M urea) = (0.22)*M ^0.52^, where *M* is the molecular mass [41]. The molecular mass for the simulated wild-type FG domain was 11,791 Daltons and for the mutant domain was 11,030 Daltons. The calculated *R*_h_ values were 22.5±4.0 for the wild-type FG domain and 28.6±5.2 for the mutant. To compare these *R*_h_ values to the Stokes radii values (*R*_s_, same as *R*_h_) for the purified FG domains in sieving columns, the contribution of a C-terminal His-tag (6 histidine residues/841 Da), which was added (post simulations) to the FG domains to aid in the purification of only full-length FG domains, had to be factored in. This was done using Uversky's scaling relationship by calculating *R*_s_ for the FG domains with the additional tag assuming a native pre-molten globular configuration for the wild-type and a native coil configuration for the mutant (see Table 1). The *R*_s_ estimated from the molecular dynamics simulations for the wild-type and mutant FG domains were multiplied by the ratio (*R*_s_ (His-tag)/ *R*_s_ (no-tag)) to yield the final values of 23.1 (±4.1) Å for the wild-type FG domain and 29.6 (±5.4) Å for the mutant FG domain reported in Table 1.

## Supporting Information

Table S1 List of 45 distance indices between the Cβ atoms of Phe or substitute Ala residues in the wild type or mutant FG domains, respectively.
(0.06 MB DOC)

Table S2 List of distance constraints obtained from the Gaussian fit to interresidue distance distribution.
(0.11 MB DOC)

Table S3 The dimensions and locations of GLFG-rich domains of nups at the NPC.
(0.07 MB DOC)

Figure S1 Plots of radii of gyration versus time of the simulated Nup116 FG domains. Radii of gyration (*R*_g_) in units of Angstroms (Å) over the last 3 ns of the MD simulations for twenty replicate simulations at 300 K for the wild-type and F>A mutant FG domains. The plots show no systematic increase or decrease in the *R*_g_ values during this time window.
(6.97 MB TIF)

Figure S2 Structural dynamics of the simulated FG domains. Autocorrelation vector of all phi and psi angles calculated with a 1 ps time step averaging over all 2800 autocorrelation windows in the last 3 ns simulations time. A separate line is plotted for each of the twenty wild-type replicates (blue lines), F>A mutant replicates (red lines), and the fibroblast growth factor 1, as reference (green line).
(2.24 MB TIF)

Figure S3 Comparison of internal structural correlation between the simulated FG domains. Back-to-back histograms of the Pearson correlation coefficients calculated between all F–F (or A–A) distance pairs measured every 1 ps during the last 3 ns of simulation time at 300 K in all 20 replicates. Note that the wild-type FG domain shows a systematic bias towards higher correlation coefficients, indicating more internal structural correlation in comparison to the F>A mutant.
(0.74 MB TIF)

Figure S4 A hydrophobic cluster analysis (HCA) of the Nup116 FG domain. (A) HCA analysis of the wild-type and F>A mutant FG domains (AA 348–458) was performed using the web server http://bioserv.impmc.jussieu.fr/hca-file.html. The AA sequences used are listed in Figure 1C. The notation for the various symbols follows the original references on HCA: a star for proline, a diamond for glycine, a square with a dot for serine, a square without a dot for threonine, and a box surrounding hydrophobic residues [50],[51].
(3.51 MB TIF)

Figure S5 The dynamic behavior of GLFG-rich domains of nucleoporins within the transport conduit of the yeast nuclear pore complex. These FG domains are depicted as a dynamic ensemble of premolten globular structures that can fluctuate widely in dimensions. Other types of FG domains are excluded for simplicity. (A) In their “ground state” the GLFG-rich domains are too small to span across the NPC transport conduit from their tether sites within the NPC scaffold, but are large enough to contact each other locally within a single NPC spoke (see top panel) and between adjacent spokes (see bottom panel), based on their close anchoring at the NPC (see Table S3). In this panel, the FG motifs are shown in equilibrium between *intra*- and *inter*molecular interactions. (B) Fluctuations in the dimensions of FG domains (which are intrinsic to natively unfolded structures) and steric hindrance effects between FG domains (due to their close anchoring) could cause the FG domains to extend further out into the transport conduit. The cohesive properties between FG domains within the conduit (and the cross-linking action of karyopherins; see below) could transiently stabilize some of the extended conformations. In this panel, the FG motifs are shown in competition between *intra*- and *inter*molecular interactions. (C) During transit across the nuclear pore complex, karyopherin-cargo complexes likely separate and bridge FG domains by interacting with their FG motifs. In this panel, the FG motifs are shown in competition between *intra*- and *inter*molecular FG motif interactions as well as in competition with karyopherins. In all panels, the nuclear envelope is shown in gray, the nuclear pore complex in green, its cytoplasmic fibrils in light yellow, its nuclear basket in light red, the GLFG-rich domains of nucleoporins in blue, and their *intra*- and *inter*molecular FG motif interactions in bright red. For panel C, karyopherins are shown in black and their cargo in bright yellow.
(1.58 MB TIF)
