# Supplementary material for: Intramolecular Cohesion of Coils Mediated by Phenylalanine–Glycine Motifs in the Natively Unfolded Domain of a Nucleoporin
Source: PLoS Comput Biol. 2008 Aug 8;4(8):e1000145. doi: 10.1371/journal.pcbi.1000145 (PMC2475668; doi:10.1371/journal.pcbi.1000145)
Supplement: Table S1 — List of 45 distance indices between the Cβ atoms of Phe or substitute Ala residues in the wild type or mutant FG domains, respectively. (0.06 MB DOC) [file pcbi.1000145.s001.doc]

| **Table S1:** List of 45 distance indices between the Cβ atoms of Phe or substitute Ala residues in the wild type or mutant FG domains, respectively. | | | | | |
| --- | --- | --- | --- | --- | --- |
| index | Distance | | index | Distance | |
|  | from Cβ | to Cβ |  | from Cβ | to Cβ |
| 1 | 13 | 23 | 24 | 32 | 112 |
| 2 | 13 | 32 | 25 | 46 | 59 |
| 3 | 13 | 46 | 26 | 46 | 71 |
| 4 | 13 | 59 | 27 | 46 | 84 |
| 5 | 13 | 71 | 28 | 46 | 93 |
| 6 | 13 | 84 | 29 | 46 | 103 |
| 7 | 13 | 93 | 30 | 46 | 112 |
| 8 | 13 | 103 | 31 | 59 | 71 |
| 9 | 13 | 112 | 32 | 59 | 84 |
| 10 | 23 | 32 | 33 | 59 | 93 |
| 11 | 23 | 46 | 34 | 59 | 103 |
| 12 | 23 | 59 | 35 | 59 | 112 |
| 13 | 23 | 71 | 36 | 71 | 84 |
| 14 | 23 | 84 | 37 | 71 | 93 |
| 15 | 23 | 93 | 38 | 71 | 103 |
| 16 | 23 | 103 | 39 | 71 | 112 |
| 17 | 23 | 112 | 40 | 84 | 93 |
| 18 | 32 | 46 | 41 | 84 | 103 |
| 19 | 32 | 59 | 42 | 84 | 112 |
| 20 | 32 | 71 | 43 | 93 | 103 |
| 21 | 32 | 84 | 44 | 93 | 112 |
| 22 | 32 | 93 | 45 | 103 | 112 |
| 23 | 32 | 103 |  |  |  |
