# Supplementary material for: Intramolecular Cohesion of Coils Mediated by Phenylalanine–Glycine Motifs in the Natively Unfolded Domain of a Nucleoporin
Source: PLoS Comput Biol. 2008 Aug 8;4(8):e1000145. doi: 10.1371/journal.pcbi.1000145 (PMC2475668; doi:10.1371/journal.pcbi.1000145)
Supplement: Table S2 — List of distance constraints obtained from the Gaussian fit to interresidue distance distribution. (0.11 MB DOC) [file pcbi.1000145.s002.doc]

| **Table S2:** List of distance constraints obtained from the Gaussian fit to inter-residue distance distribution. | | | | | | | | | | | | | | | | | |
| --- | --- | --- | --- | --- | --- | --- | --- | --- | --- | --- | --- | --- | --- | --- | --- | --- | --- |
| Gaussian fit results of distances (Å) | | | | | | | | | Gaussian fit results of distances (Å) | | | | | | | | |
| Index | wild type FG domain | | | | mutant (F>A) FG domain | | | | Index | wild type FG domain | | | | mutant (F>A) FG domain | | | |
|  | Phe | Phe | Center | Width | Ala | Ala | Center | Width |  | Phe | Phe | Center | Width | Ala | Ala | Center | Width |
| 9 | 13 | 23 | 13.8 | 4.8 | 13 | 23 | 13.9 | 4.7 | 16 | 32 | 112 | 20.6 | 10.6 | 32 | 112 | 25.1 | 11.1 |
| 17 | 13 | 32 | 17.2 | 7.5 | 13 | 32 | 20.8 | 6.6 | 18 | **46** | **59** | **13.8** | **4.4** | **46** | **59** | **15.5** | **5.6** |
| 24 | 13 | 46 | 19.4 | 7.5 | 13 | 46 | 27.2 | 10.0 | 19 | 46 | 71 | 20.4 | 7.7 | **46** | **71** | **20.5** | **10.0** |
| 30 | **13** | **59** | **20.8** | **10.4** | 13 | 59 | 25.3 | 12.9 | 20 | 46 | 84 | 23.0 | 9.3 | **46** | **84** | **30.8** | **10.0** |
| 35 | 13 | 71 | 21.6 | 8.7 | 13 | 71 | 20.2 | 5.9 | 21 | 46 | 93 | 25.0 | 7.9 | 46 | 93 | 30.2 | 14.2 |
| 39 | **13** | **84** | **17.6** | **6.3** | 13 | 84 | 14.9 | 6.0 | 22 | 46 | 103 | 25.9 | 6.8 | 46 | 103 | 31.5 | 10.9 |
| 42 | 13 | 93 | 16.2 | 9.5 | 13 | 93 | 17.9 | 4.9 | 23 | 46 | 112 | 25.5 | 7.0 | 46 | 112 | 30.4 | 10.8 |
| 44 | 13 | 103 | 16.0 | 5.9 | 13 | 103 | 15.7 | 6.1 | 25 | **59** | **71** | **14.5** | **5.6** | **59** | **71** | **15.7** | **6.4** |
| 45 | **13** | **112** | **16.2** | **6.0** | **13** | **112** | **18.0** | **7.1** | 26 | **59** | **84** | **18.0** | **5.6** | 59 | 84 | 25.3 | 8.4 |
| 1 | **23** | **32** | **15.0** | **6.9** | **23** | **32** | **14.3** | **3.3** | 27 | 59 | 93 | 23.4 | 9.2 | 59 | 93 | 26.1 | 14.1 |
| 2 | 23 | 46 | 20.4 | 8.0 | **23** | **46** | **22.1** | **5.7** | 28 | **59** | **103** | **27.9** | **9.8** | 59 | 103 | 28.7 | 8.4 |
| 3 | 23 | 59 | 21.4 | 7.9 | 23 | 59 | 21.6 | 9.7 | 29 | **59** | **112** | **25.6** | **15.8** | 59 | 112 | 31.8 | 13.4 |
| 4 | 23 | 71 | 20.2 | 10.0 | 23 | 71 | 21.4 | 12.9 | 31 | **71** | **84** | **16.3** | **10.2** | **71** | **84** | **19.7** | **7.1** |
| 5 | 23 | 84 | 20.5 | 9.6 | 23 | 84 | 24.1 | 13.3 | 32 | 71 | 93 | 24.1 | 8.8 | 71 | 93 | 22.1 | 9.8 |
| 6 | **23** | **93** | **20.8** | **10.5** | 23 | 93 | 21.9 | 11.5 | 33 | 71 | 103 | 25.2 | 9.1 | 71 | 103 | 26.9 | 9.6 |
| 7 | 23 | 103 | 19.8 | 8.3 | **23** | **103** | **18.4** | **5.7** | 34 | **71** | **112** | **24.4** | **10.4** | 71 | 112 | 26.2 | 9.6 |
| 8 | 23 | 112 | 20.6 | 7.4 | 23 | 112 | 21.9 | 11.3 | 36 | **84** | **93** | **14.2** | **4.7** | 84 | 93 | 24.4 | 4.3 |
| 10 | **32** | **46** | **16.0** | **7.0** | **32** | **46** | **17.4** | **5.0** | 37 | 84 | 103 | 18.4 | 9.9 | 84 | 103 | 19.9 | 8.8 |
| 11 | 32 | 59 | 16.5 | 7.3 | **32** | **59** | **21.5** | **6.8** | 38 | **84** | **112** | **20.5** | **9.3** | 84 | 112 | 22.9 | 9.4 |
| 12 | 32 | 71 | 20.8 | 9.3 | 32 | 71 | 23.8 | 11.2 | 40 | **93** | **103** | **14.3** | **5.7** | **93** | **103** | **15.3** | **3.6** |
| 13 | 32 | 84 | 19.7 | 7.0 | 32 | 84 | 28.3 | 12.6 | 41 | **93** | **112** | **21.0** | **7.2** | 93 | 112 | 21.7 | 4.6 |
| 14 | 32 | 93 | 22.3 | 7.7 | 32 | 93 | 26.1 | 16.4 | 43 | **103** | **112** | **13.7** | **3.6** | **103** | **112** | **13.8** | **3.9** |
| 15 | 32 | 103 | 24.0 | 12.2 | 32 | 103 | 23.7 | 13.9 |  |  |  |  |  |  |  |  |  |
