# Supplementary material for: Intramolecular Cohesion of Coils Mediated by Phenylalanine–Glycine Motifs in the Natively Unfolded Domain of a Nucleoporin
Source: PLoS Comput Biol. 2008 Aug 8;4(8):e1000145. doi: 10.1371/journal.pcbi.1000145 (PMC2475668; doi:10.1371/journal.pcbi.1000145)
Supplement: Table S3 — The dimensions and locations of GLFG-rich domains of nups at the NPC. (0.07 MB DOC) [file pcbi.1000145.s003.doc]

| **Table S3** | |  | |  | |  | |  | |  | |  | |  |  |  |
| --- | --- | --- | --- | --- | --- | --- | --- | --- | --- | --- | --- | --- | --- | --- | --- | --- |
|  |  |  | |  | |  | |  | |  | |  | |  |  |  |
|  |  | predicted diameter of | | NPC anchor site | | NPC anchor site | | NPC anchor site | | NPC anchor site | |  | | Sed. Coeff.§ | Stokes radii § | Calculated |
|  | MW of FG domain | FG domain with flanking | | r = radius | | z = height | | r = radius | | z = height | | MW of | | in Svedbergs | Rs or Rh | diameter |
|  | with flanking | disordered regions¥ | | from NPC conduit center | | from NPC midplane at NE | | from NPC conduit center | | from NPC midplane at NE | | full-length nup | | for full-length nup | for full-length nup | for full-length nup |
| FG nup | disordered regions¥ | assuming a PMG configuration | | from immuno-EM data† | | from immuno-EM data† | | from Modeller data† | | from Modeller data† | | with Protein A tag¶ | | with Protein A tag¶ | with Protein A tag¶ | with Protein A tag¶ |
|  |  |  | |  | |  | |  | |  | |  | |  |  |  |
| Nup116 | 99000 Da | 12.0 nm | | 30 ± 5 nm | | 11 ± 4 nm | | 27 nm | | 13 nm | | 100000 Da | | 3.6 ± 0.8 S | 7.15 nm | 14.3 nm |
| Nup100 | 82000 Da | 11.1 nm | | 28 ± 5 nm | | 8 ± 4 nm | | 22 nm | | 10 nm | | 126000 Da | | 4.4 ± 0.8 S | 7.37 nm | 14.7 nm |
| Nup49 | 24300 Da | 6.8 nm | | 25 ± 5 nm | | 7 ± 3 nm | | 29 nm | | 4 nm | | 75000 Da | | 3.9 ± 0.8 S | 4.95 nm | 9.9 nm |
| Nup57 | 24600 Da | 6.8 nm | | 19 ± 11 nm | | -3 ± 5 nm | | 29 nm | | 4 nm | | 83000 Da | | 4.1 ± 0.8 S | 5.21 nm | 10.4 nm |
| Nup145n | 21300 Da | 6.5 nm | | 26 ± 14 nm | | -11± 6 nm | | 32 nm | | -14, -5 nm | | 86000 Da | | 3.7 ± 0.8 S | 5.98 nm | 12.0 nm |
|  |  |  | |  | |  | |  | |  | |  | |  |  |  |
| ¥ Includes FG domain plus flanking sequences that are predicted to be intrinsically unstructured, as delineated in Denning & Rexach, 2007 | | | | | | | | | |  | |  | |  |  |  |
| † Data from Alber et al 2007a, 2007b. | | |  | |  | |  | | |  | |  | |  |  |  |
| ¶ Includes a 26 kDa Protein A tag in addition to the full-length Nup sequence (data from Alber et al 2007a). | | | | | | |  | | |  | |  | |  |  |  |
| § These values were obtained directly from Alber et al 2007, or were mathematically-derived using their published MW and sedimentation coefficient values and the conversion formula used in Denning et al 2002. | | | | | | | | | | | | | | |  |  |
|  |  |  |  | |  | |  | |  | |  | |  | |  |  |
