# Supplementary figures and images for: Intramolecular Cohesion of Coils Mediated by Phenylalanine–Glycine Motifs in the Natively Unfolded Domain of a Nucleoporin
Source: PLoS Comput Biol. 2008 Aug 8;4(8):e1000145. doi: 10.1371/journal.pcbi.1000145 (PMC2475668; doi:10.1371/journal.pcbi.1000145)

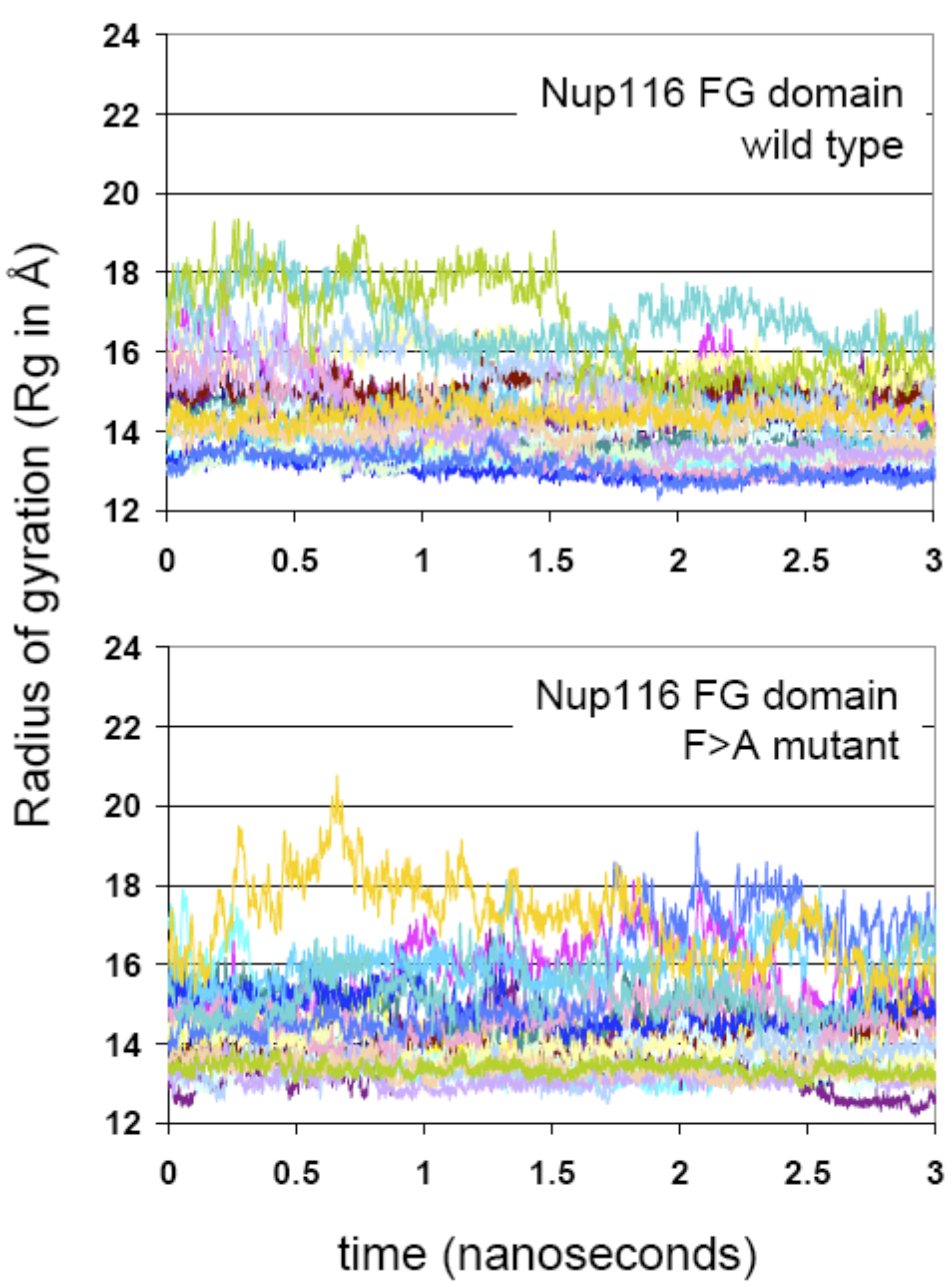

Supplement: Figure S1 — Plots of radii of gyration versus time of the simulated Nup116 FG domains. Radii of gyration (R g) in units of Angstroms (Å) over the last 3 ns of the MD simulations for twenty replicate simulations at 300 K for the wild-type and F>A mutant FG domains. The plots show no systematic increase or decrease in the R g values during this time window. (6.97 MB TIF) [file pcbi.1000145.s004.tif]

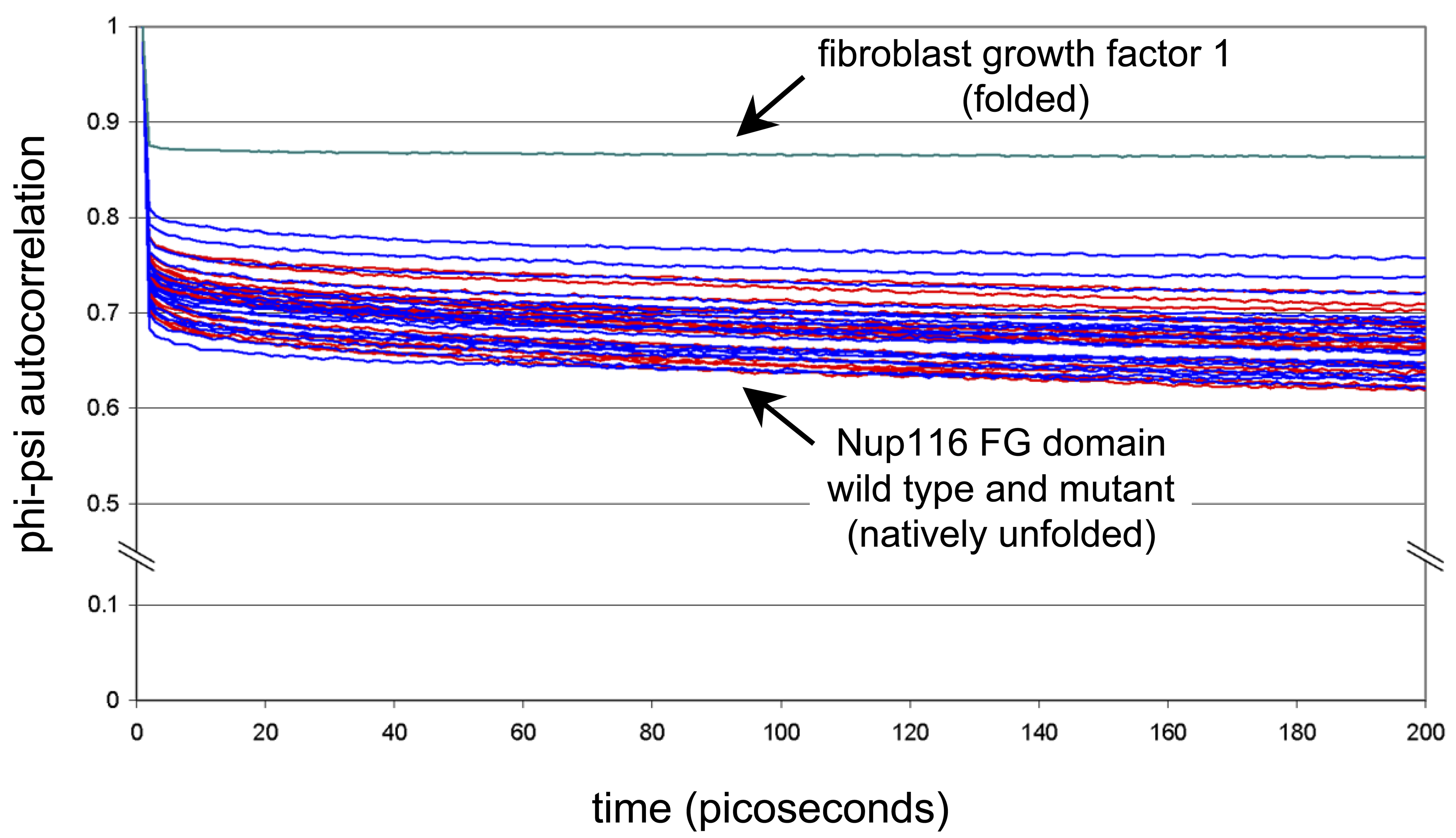

Supplement: Figure S2 — Structural dynamics of the simulated FG domains. Autocorrelation vector of all phi and psi angles calculated with a 1 ps time step averaging over all 2800 autocorrelation windows in the last 3 ns simulations time. A separate line is plotted for each of the twenty wild-type replicates (blue lines), F>A mutant replicates (red lines), and the fibroblast growth factor 1, as reference (green line). (2.24 MB TIF) [file pcbi.1000145.s005.tif]

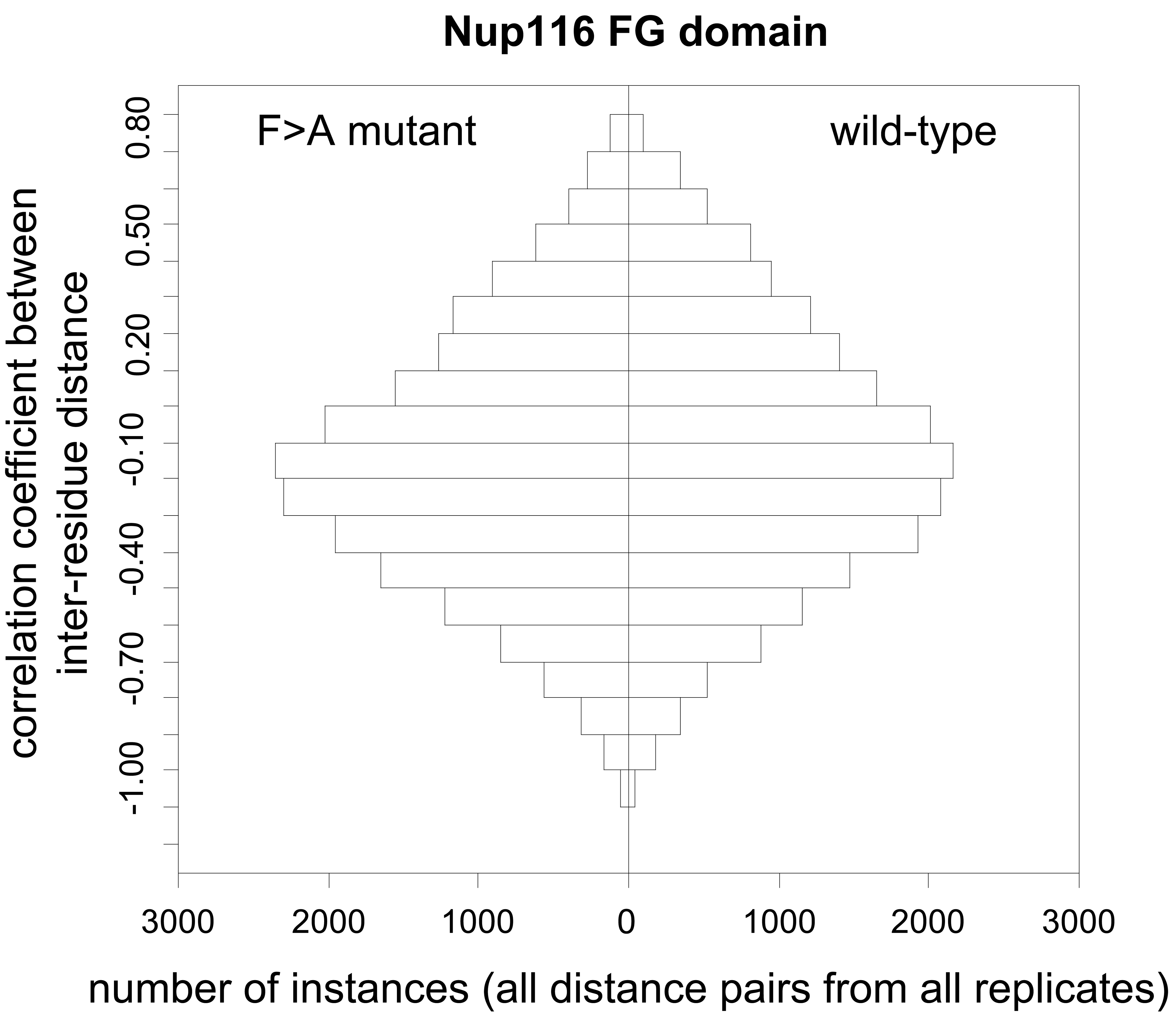

Supplement: Figure S3 — Comparison of internal structural correlation between the simulated FG domains. Back-to-back histograms of the Pearson correlation coefficients calculated between all F–F (or A–A) distance pairs measured every 1 ps during the last 3 ns of simulation time at 300 K in all 20 replicates. Note that the wild-type FG domain shows a systematic bias towards higher correlation coefficients, indicating more internal structural correlation in comparison to the F>A mutant. (0.74 MB TIF) [file pcbi.1000145.s006.tif]

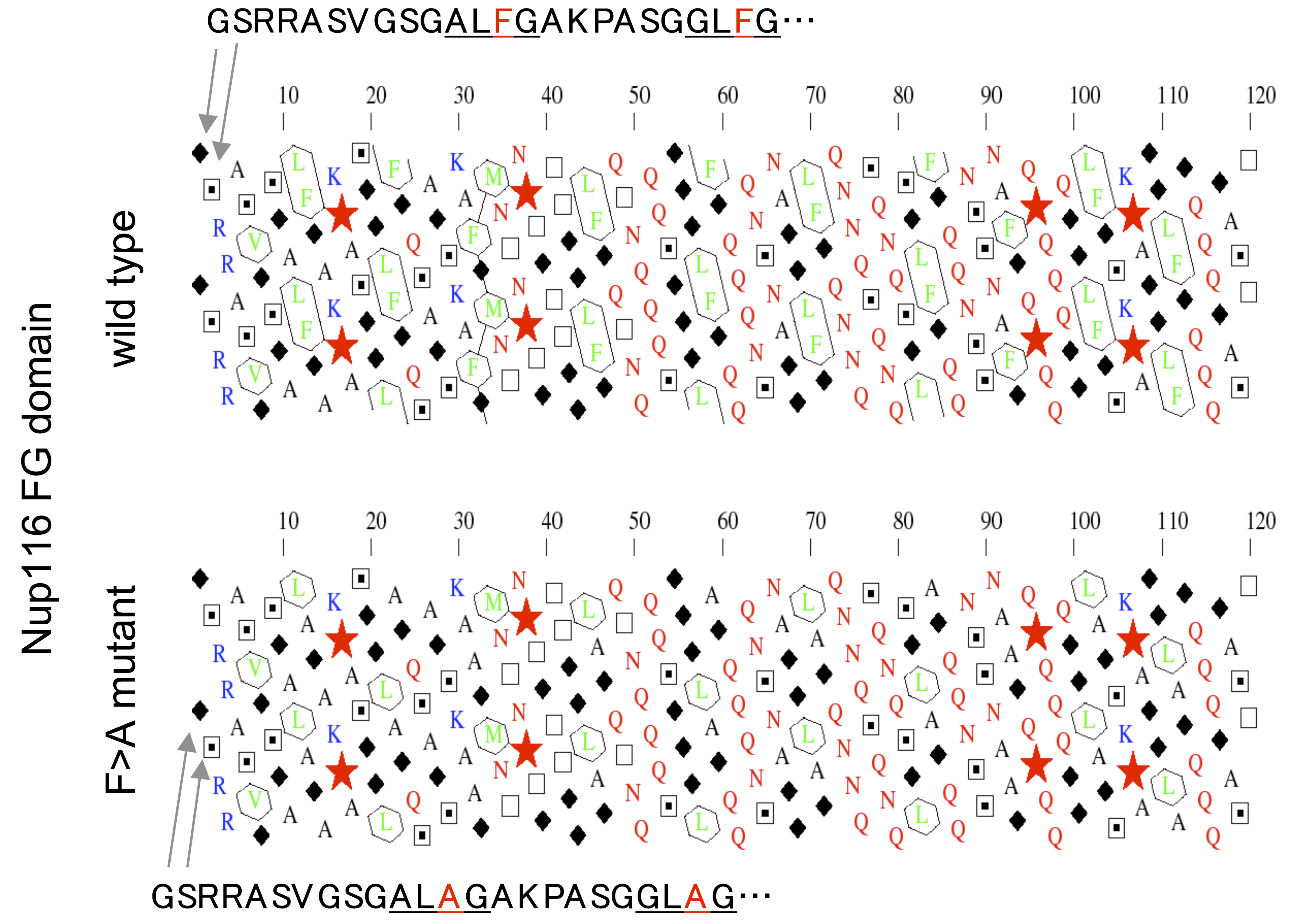

Supplement: Figure S4 — A hydrophobic cluster analysis (HCA) of the Nup116 FG domain. (A) HCA analysis of the wild-type and F>A mutant FG domains (AA 348–458) was performed using the web server http://bioserv.impmc.jussieu.fr/hca-file.html. The AA sequences used are listed in Figure 1C. The notation for the various symbols follows the original references on HCA: a star for proline, a diamond for glycine, a square with a dot for serine, a square without a dot for threonine, and a box surrounding hydrophobic residues [50],[51]. (3.51 MB TIF) [file pcbi.1000145.s007.tif]

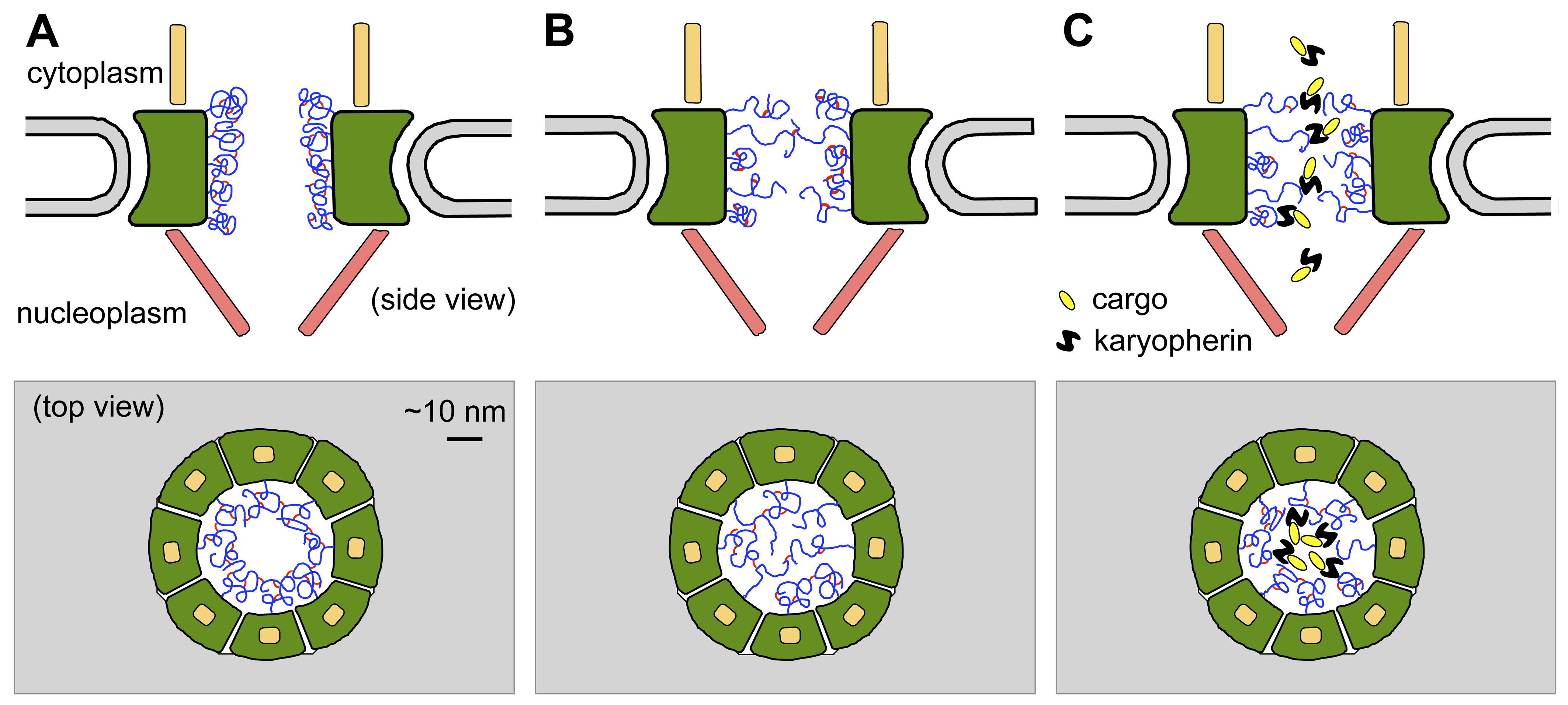

Supplement: Figure S5 — The dynamic behavior of GLFG-rich domains of nucleoporins within the transport conduit of the yeast nuclear pore complex. These FG domains are depicted as a dynamic ensemble of premolten globular structures that can fluctuate widely in dimensions. Other types of FG domains are excluded for simplicity. (A) In their “ground state” the GLFG-rich domains are too small to span across the NPC transport conduit from their tether sites within the NPC scaffold, but are large enough to contact each other locally within a single NPC spoke (see top panel) and between adjacent spokes (see bottom panel), based on their close anchoring at the NPC (see Table S3). In this panel, the FG motifs are shown in equilibrium between intra- and intermolecular interactions. (B) Fluctuations in the dimensions of FG domains (which are intrinsic to natively unfolded structures) and steric hindrance effects between FG domains (due to their close anchoring) could cause the FG domains to extend further out into the transport conduit. The cohesive properties between FG domains within the conduit (and the cross-linking action of karyopherins; see below) could transiently stabilize some of the extended conformations. In this panel, the FG motifs are shown in competition between intra- and intermolecular interactions. (C) During transit across the nuclear pore complex, karyopherin-cargo complexes likely separate and bridge FG domains by interacting with their FG motifs. In this panel, the FG motifs are shown in competition between intra- and intermolecular FG motif interactions as well as in competition with karyopherins. In all panels, the nuclear envelope is shown in gray, the nuclear pore complex in green, its cytoplasmic fibrils in light yellow, its nuclear basket in light red, the GLFG-rich domains of nucleoporins in blue, and their intra- and intermolecular FG motif interactions in bright red. For panel C, karyopherins are shown in black and their cargo in bright yellow. (1.58 MB TIF) [file pcbi.1000145.s008.tif]
